# Multifaceted Mechanisms of WY-14643 to Stabilize the Blood-Brain Barrier in a Model of Traumatic Brain Injury

**DOI:** 10.3389/fnmol.2017.00149

**Published:** 2017-05-26

**Authors:** Winfried Neuhaus, Tobias Krämer, Anja Neuhoff, Christina Gölz, Serge C. Thal, Carola Y. Förster

**Affiliations:** ^1^Competence Unit Molecular Diagnostics, Competence Center Health and Bioresources, AIT Austrian Institute of Technology (AIT) GmbHVienna, Austria; ^2^Department of Anesthesiology, Medical Center of Johannes Gutenberg University of MainzMainz, Germany; ^3^Department of Anesthesia and Critical Care, Center of Operative Medicine, University Hospital WürzburgWürzburg, Germany

**Keywords:** pirinixic acid, PPARα, stroke, traumatic brain injury, ischemia, blood-brain barrier

## Abstract

The blood-brain barrier (BBB) is damaged during ischemic insults such as traumatic brain injury or stroke. This contributes to vasogenic edema formation and deteriorate disease outcomes. Enormous efforts are pursued to understand underlying mechanisms of ischemic insults and develop novel therapeutic strategies. In the present study the effects of PPARα agonist WY-14643 were investigated to prevent BBB breakdown and reduce edema formation. WY-14643 inhibited barrier damage in a mouse BBB *in vitro* model of traumatic brain injury based on oxygen/glucose deprivation in a concentration dependent manner. This was linked to changes of the localization of tight junction proteins. Furthermore, WY-14643 altered phosphorylation of kinases ERK1/2, p38, and SAPK/JNK and was able to inhibit proteosomal activity. Moreover, addition of WY-14643 upregulated PAI-1 leading to decreased t-PA activity. Mouse *in vivo* experiments showed significantly decreased edema formation in a controlled cortical impact model of traumatic brain injury after WY-14643 application, which was not found in PAI-1 knockout mice. Generally, data suggested that WY-14643 induced cellular responses which were dependent as well as independent from PPARα mediated transcription. In conclusion, novel mechanisms of a PPARα agonist were elucidated to attenuate BBB breakdown during traumatic brain injury *in vitro*.

## Introduction

Cerebral ischemic insults represent an immense burden for national health care systems. The total European annual health care cost of traumatic brain injury (TBI) is over 33 billion € (Berg et al., 2005). The incidence of cranio-cerebral traumas in industrialized countries is 200–300 per 100,000 humans per year (Baethmann et al., 2002). It was estimated that every year 2.5 million people sustain a TBI leading to 53,000 TBI-related deaths in the United States (Frieden et al., 2014).

Traumatic brain injury mediated damages of the brain proceed in two phases. Initial direct mechanical impact such as brain contusion causes parenchymal damage, which becomes evident as tissue destruction, axonal shearing, and hemorrhage associated with increased cerebrovascular permeability and impaired cerebral blood flow (Thal and Neuhaus, 2014). Within hours to days the primary brain damage induces a set of secondary processes on the cellular, metabolic, and molecular level such as oxidative stress, inflammation, and apoptosis. These events could cause further cerebrovascular disruption and neuronal loss leading, eventually, to disability or even death (Verma, 2000; Cernak, 2005; Finnie, 2014; Logsdon et al., 2015; Su et al., 2015). Cerebral edema formation is one of the most serious and difficult to control secondary effects of TBI and one of the main factors for the high mortality and morbidity after TBI. The causes of edema in TBI patients are complex, but it is well appreciated that leakage of the blood-brain barrier (BBB) induced by cerebral ischemia or hypoxia is a significant factor in the development of vasogenic edema (Atlee, 2007; Chodobski et al., 2011; Gray, 2011).

Several therapeutic strategies for TBI have been developed in the last decades. However, numerous animal studies and clinical trials were unsuccessful, and no effective therapy for TBI patients has emerged (Grumme et al., 1995; Marmarou et al., 1999; Bramlett and Dietrich, 2004; Yurkewicz et al., 2005; Maas et al., 2006, 2010; Xiong et al., 2009; Su et al., 2015). Therefore, the therapeutic focus is still set on activities to reduce brain edema and intracranial pressure (Thal and Neuhaus, 2014).

In the development of novel therapeutic approaches it could be useful to evaluate treatment strategies for other diseases with similar events during the disease. In the case of TBI and cerebral edema, the comparison with stroke seems to be meaningful. Although the primary cause of TBI and stroke are substantially different, the secondary mechanisms of brain infarction are overlapping. In particular, excitotoxicity, inflammation, free radical damage, and BBB disruption are hallmarks of both TBI and stroke (Sashindranath et al., 2011). In case of stroke current treatments are only based on thrombolysis by, e.g., tissue plasminogen activator (t-PA) in the acute phase of stroke. Another approach to decrease the number of cerebral ischemic insults is to minimize risk factors. The most important risk for stroke is high blood pressure. In addition, high concentrations of lipids in blood have been identified as another main risk factor (Lawes et al., 2004; Amarenco and Labreuche, 2009). In recent years, it was hypothesized that low triglycerides, high HDL levels or lower LDL/HDL ratios were more protective than low concentrations of total cholesterol or LDL-bound cholesterol (Labreuche et al., 2010; Deplanque and Amarenco, 2011). Clinical studies showed that lipid-lowering compounds were able to reduce the number of fatal stroke, but not the incidence of stroke (Zhou et al., 2013). Therefore, it is believed that lipid-lowering substances such as peroxisome proliferator-activated receptor (PPAR) agonists are able to attenuate stroke damage. Some studies reported that the combination of statins with PPAR agonists show additional beneficial effects on adverse stroke outcome, especially in cases of high risk groups with, e.g., atherogenic dyslipidemia (Montaner et al., 2008; van der Most et al., 2009; Castilla-Guerra and Fernandez-Moreno Mdel, 2015).

Peroxisome proliferator-activated receptors are divided into the three subtypes α, β/δ, and γ, whereby especially PPARα activation leads to a decrease of lipids. PPARs belong to the group of orphan nuclear receptors which regulate DNA transcription after dimerization with retinoic acid-X-receptors (RXR) (Fruchart et al., 1999). Several studies revealed that PPAR agonists were not only beneficial via their lipid-lowering effects. They additionally exerted pleiotropic mechanisms modulating inflammatory, immune and oxidative pathways (Staels et al., 1998; Delerive et al., 1999; Escher and Wahli, 2000; Von Knethen and Brüne, 2001; Cunard et al., 2002a,b). Since PPARs are expressed in neurons as well as in glial and endothelial cells, PPAR agonists were not only neuroprotective, they also reduced microglial activation or BBB permeability (Ouk et al., 2009; Gautier et al., 2015). This could be of immense relevance, because clinical studies focusing only on neuroprotection were not successful in the last decades indicating that future therapeutic strategies should be extended toward additional target cells (Turner et al., 2013).

In this regard the term neurovascular unit describes the fact that the functionality of the BBB is highly regulated by its microenvironment, and that also brain capillary endothelial cells could regulate the functionality of CNS cells as well. That’s why microglial cells as well as brain endothelial cells got into the focus of recent research about novel targets. Indeed, the breakdown of the BBB contributes to vasogenic edema formation during ischemic insults and the stabilization of the BBB could reduce edema formation and brain tissue damage (Lo et al., 2001; Abbott et al., 2006; Neuwelt et al., 2008; Abbott and Friedman, 2012). Brain capillary endothelial cells are distinctly different compared to peripheral endothelial cells. Their intercellular gaps are sealed by tight junctions, the pinocytotic activity is remarkably decreased and they do not form fenestrae (Joó, 1996). In addition, they possess a significant array of active transporter proteins and exhibit an increased metabolic activity. All these features contribute to the barrier function which is generally comprised by its physical (paracellular), transporter and metabolic components (Neuhaus and Noe, 2009).

*In vivo* as well as *in vitro* studies confirmed that preventive treatment, administration during the acute phase as well as therapeutic application of PPARα agonists such as fenofibrate could decrease brain damage after cerebral ischemia (Ouk et al., 2009, 2014; Gautier et al., 2015). It was shown that treatment during the acute phase was necessary to prevent BBB breakdown and that this was dependent on the presence of PPARα, because fibrate medications were not effective in PPARα knock-out mice or in models based on brain endothelial cells derived from PPARα knock-out mice (Inoue et al., 2003; Mysiorek et al., 2009; Guo et al., 2010). With regard to TBI, therapy with fenofibrate in a TBI-rat model revealed also beneficial effects on the adverse outcomes (Besson et al., 2005; Chen et al., 2007). However, despite these efforts the underlying BBB protecting mechanisms are still not clarified.

In this regard, the choice of an appropriate *in vitro* model to simulate ischemia induced BBB breakdown is of pivotal importance. A huge array of *in vitro* models for TBI exists comprising of different scratch, strain/stretch or organotypic hippocampal slice models (Morrison et al., 1998; Thal and Neuhaus, 2014). Most models are simplified and are not capable to include or handle all relevant parts during a brain injury. To investigate the ischemic component, cells in these models are treated by hypoxia or oxygen/glucose deprivation (OGD). This could be applied alone or in combination with mechanical stress models (Thal and Neuhaus, 2014). Recently, we have analyzed the effects of stretch, OGD and the combination of stretch with OGD on brain endothelial cells as a TBI *in vitro* model of the BBB. Results revealed that the effects of OGD were significantly more prominent than the influence of stretch. Moreover, only OGD led to a distinct breakdown of the paracellular barrier of brain endothelial cell layers (Salvador et al., 2015). In addition, measurement of the transendothelial electrical resistance (TEER) as important tightness parameter was only applicable in the Transwell model, but not in the stretch or in the combined stretch/OGD-set-up. Based on these facts it was decided to investigate the effects of PPARα agonist WY-14643 in our recently validated *in vitro* BBB OGD-model (Neuhaus et al., 2014).

Our data describe for the first time underlying molecular mechanisms of PPARα agonist WY-14643 for the stabilization of the BBB under ischemic conditions. In correspondence to the literature, we show two types of effects. On the one hand we found inhibition of functional barrier breakdown determined by TEER and fluorescein permeability, alteration of tight junction protein localization and changes of enzyme activities (MAP kinases, proteasomal enzymes). These effects were not inhibited by PPARα antagonist GW6471. On the contrary, we found that WY-14643 upregulated the expression of plasminogen activator inhibitor-1 (PAI-1) in brain endothelial cells for the first time. This was blocked by PPARα antagonist GW6471 and led to decreased t-PA activity. Mouse experiments confirmed beneficial effects of WY-14643 in a TBI model. Data supported that WY-14643 acted via pathways which either were or were not inhibited by PPARα antagonist GW6471. This opens the discussion about probably involved cellular processes which require the presence of PPARα, but not its direct transcriptional activity.

## Materials and Methods

### Material

Collagen IV from human placenta (C5533), PBS (D8537), Triton-X 100 (T8787), DMEM (D5796), DAPI (D8417), β-mercapto-ethanol (M6250), fluorescein sodium (F6377), *N*-Succinyl-Leu-Leu-Val-Tyr-7-amido-4-methylcoumarin (S6510), Boc-Leu-Ser-Thr-Arg-7-amido-4-methylcoumarin (B4636), Z-Leu-Leu-Glu-7-amido-4-methylcoumarin (C0483), MG132 (C2211, Z-Leu-Leu-Leu-al), Lactacystin (L6785), WY-14643 (C7081), GW6471 (G5045), rhPAI-1 (A8111), albumin from bovine serum for immunofluorescence microscopy (fraction V, A9647) and for western blotting (A7906) were purchased from Sigma–Aldrich. DMEM without glucose (11966-025, Gibco^®^) was obtained from Life technologies (USA). FCS Gold EU approved was bought from PAA Laboratories (A15151, Lot A15111-2018, Linz, Austria) and was heat-inactivated in a water-bath at 56°C for 30 min. Penicillin/streptomycin (100X, 10,000 Units/mL, 10,000 μg/mL, A2213) and 0.05% Tyrpsin/0.02% EDTA-solution (L2143) were from BioChrom AG (Berlin, Germany). 6-well and 24-well plates and 24-well Transwell^®^ inserts (0.4 μm pore size, PET) were obtained from Becton and Dickinson (REF353046, REF353035, REF353226, USA). Gelatine was from SERVA (22151, Heidelberg, Germany), nuclease-free water was purchased from Ambion (AM9937, USA). All other substances were of analytical grade.

### Cell Culture

Mouse brain endothelial cell line cerebEND was cultured in DMEM medium supplemented with 10% FCS and 1% penicillin/streptomycin in 0.5% gelatine coated cell culture tissue flasks, rat glioma cell line C6 (ATCC) was grown with the same culture medium also in 0.5% gelatine coated tissue flasks (Silwedel and Förster, 2006). Cells were maintained in an incubator at 37°C, 95% humidity and a 5% CO2/95% air atmosphere and subcultivated 1:3 or 1:20, respectively, once a week (Neuhaus et al., 2012a). Transwell experiments were accomplished as recently reported (Neuhaus et al., 2014). In brief, cerebENDs were cultivated on collagen-IV coated 24-well inserts, on day 9 inserts with cerebENDs were placed over C6 cell containing well-plates to form the co-culture set-up. OGD experiments were conducted on day 13. After washing steps cells were subjected to serum-free DMEM with or without glucose supplemented with the substances. All solutions had the same end-concentrations of solvents. TEER was measured using chopstick electrodes from Millipore after 30 min of equilibration at RT as previously published (Neuhaus et al., 2006, 2014). After 4 h OGD treatment (1% O_2_) TEER was determined followed by a transport study with 10 μM fluorescein. TEER [Ohm × cm^2^] and permeability coefficients Pe including blank insert values were calculated according the clearance principle as previously reported (Neuhaus et al., 2008; Novakova et al., 2014). Due to comparison reasons, TEER values of normoxic mono-cultured cerebEND controls and permeability coefficients of OGD-treated co-cultures (OC6) were set to 100%.

### Quantitative Polymerase Chain Reaction (qPCR)

For quantitative polymerase chain reaction (qPCR) experiments cerebEND and C6 cells were cultivated on 6-well plates. NC6 is the abbreviation for normoxia-treated cerebEND cells with the supernatant of normoxic treated C6 cells, OC6 means OGD-treated cerebEND cells with the supernatant of OGD-treated C6 cells. In these cases C6 cells were incubated for 4 h normoxia or OGD to obtain their supernatants which were directly applied on PBS washed cerebEND cell layers to guarentee fresh soluble factors of C6 cells for further 4 h incubation. RNA isolation, cDNA transcription and qPCR were accomplished as recently published (Neuhaus et al., 2014). Used FAM-labeled probes from Taqman^®^ (Applied Biosystems) were listed in Supplementary Table S1. In case of PPARα and LRP1, cDNA was preamplified using Taqman PreAmp Master mix (2x) prior qPCR analysis according to Neuhaus et al. (2012a, Supplementary Part). Each sample was analyzed as triplicate. Relative mRNA abundances to β-actin were calculated by the ddCt method using following formula: 2^(Ct of β-actin–Ct of gene of interest)^, where Ct is the threshold cycle value.

### Western Blotting

Western blotting was conducted as recently published (Neuhaus et al., 2014). In brief, cells were scraped after the treatments in RIPA buffer on ice. In case of membran protein enrichment, proteins were extracted with 1% Triton-X 100 followed by lysis of the residual proteins in RIPA buffer. All lysis buffers were supplemented with protease inhibitor cocktail and PhosphoSTOP. Protein concentrations were measured by a detergent-compatible Pierce BCA assay (Fisher Scientific). Twenty microgram protein of total lysates or 10 μg of Triton-X 100 and RIPA fractions per lane were loaded onto 7.5, 10, or 12% SDS–PAGE gels. After gel electrophoresis proteins were immunoblotted onto polyvinylidene difluoride membranes. Incubations with primary and secondary antibodies (Supplementary Table S2) were carried out as previously described (Neuhaus et al., 2012b). ECL-solutions were used for the visualization of the bands using a FluorChem FC2 Multiimager II (Alpha Innotech, Hessisch Oldendorf, Germany). Density values of single protein bands were calculated with the software Alpha View and were related to the corresponding β-actin bands.

### Proteasomal Activity

Proteasomal activities were measured according to Huang et al. (2009) with slight modifications. Stock solutions of proteasomal enzyme substrates *N*-succinyl-Leu- Leu-Val-Tyr-7-amido-4-methylcoumarin (5mM, chymotrypsin-like), Boc-Leu-Ser-Thr-Arg-7-amido-4-methylcoumarin (5mM, trypsin-like), Z-Leu- Leu-Glu-7-amido-4-methylcoumarin [5mM, peptidylglutamyl-peptide hydrolyzing activity (PGPH)] as well as proteasomal inhibitors lactacystin (5mM) and MG132 (50mM) were prepared in DMSO. cerebEND and C6 cells were seeded on gelatin coated 6-wells, cultivated for 6 days and subjected to normoxia or OGD treatments as recently published (Neuhaus et al., 2014). After the treatments cells were washed with ice-cold PBS twice and lysed with 300 μL proteasome activity buffer [PAB; 10 mM Tris-HCl (pH = 7.8), 1 mM EDTA, 0.5 mM dithiothreitol, 0.5% Triton X-100, 5 mM MgCl_2_] per 6-well on ice with moderate shaking (52 rpm) for 30 min. While supernatants were stored on ice, protein concentrations were determined by BCA protein assay kit from Pierce (Cat. No. 23227) using 25 μL sample per 96-well. Then, volume for 50 μg protein per sample was supplemented with according volumes of WY-14643 and GW6471 stock solutions in DMSO and filled up with PAB to 196 μL per well to reach a total DMSO concentration of 0.4% in all samples. Four microliter enzyme substrate stock solution per well were added on ice, and the reaction started by putting the black 96-well plate in a prewarmed microplate reader (GeniosPro, Tecan, Austria) at 37°C. Fluorescence increase was recorded at 360 nm/460 nm every 10 min for 2 h. Control blank values were substracted from cell lysate values and slopes between 10 and 120 min were calculated after linear regression analysis. Finally, slopes of control lysates set to 100%. Each sample was measured as duplicate from three independent conducted experiments.

### MMP and t-PA Activity

Enzymatic activity of matrix metalloproteinases (MMP) or of t-PA of concentrated medium supernatants were measured by using 520 MMP FRET substrate SB-14 (Anaspec, USA) or component A of Sensolyte AMC t-PA activity assay kit (72160, Anaspec, USA) as recently described (Neuhaus et al., 2014). Increasing fluorescence was recorded over 120 min at 37°C in a microplate reader (GeniosPro, Tecan, Austria). Data between 10 and 120 min were used to calculate the slope by linear regression analysis. Slopes of media supernatants of OC6-treated cerebEND cells were set to 100%.

### Animals

All animal procedures were conducted in compliance with the institutional guidelines of the Johannes Gutenberg-University, Mainz, Germany and approved by the State Agency for Consumer and Health Protection (approval number: G12-1-041), and performed in accordance with the German Animal Welfare Act. The group sizes of the study were calculated prior to approval with the analysis of variance sample size. 16.2 ± 7.7 weeks old 48 male C57Bl6/N mice (Charles River Laboratories, Sulzfeld, Germany; 26.4 ± 8.3 g) and 26 weeks old 20 PA-1 deficient mice (B6.129S2-*Serpine1^tm1Mlg^*/J) with 26–32 g weight, purchased from Jackson Laboratories (Bar Harbor, ME, US), were included in the study. The compliance with the ARRIVE guidelines is confirmed. Animals were held in scantainer ventilated cabinets with exercise wheels and plastic houses at the animal facility of the Department of Experimental Surgery. The animals were exposed to a 12-h dark/light interval and ingested H_2_O and feed *ad libitum.*

### Traumatic Brain Injury

Animals were anesthetized with 5 mg/kg midazolam (Siegfried Hameln, Hameln, Germany), 0.05 mg/kg fentanyl (Merck, Darmstadt, Germany), and 0.5 mg/kg medetomidine (Zoetis, Kirkland, QC, Canada) intraperitoneally. Rectal temperature was maintained at 37°C with a thermostatically regulated, feedback-controlled heating pad (Hugo Sachs, March-Hugstetten, Germany). The TBI was induced as described before (Thal et al., 2008). The cranium was fixed in a stereotactical frame, a craniotomy was generated between lambdoid, sagittal and coronal sutures with a saline cooled high speed drill. The trauma was induced with an electromagnetic cortical impact device (Impact OneTM Stereotaxic Impactor, Richmond, IL, USA) diameter of the impactor tip: 3 mm; impact velocity: 6 m/sec; impact duration: 200 msec, and displacement: 1.5 mm. The craniotomy was closed with the initially removed bone flap using conventional tissue glue (Histoacryl, Braun-Melsungen, Germany). The skin was carefully closed with four single button sutures, anaesthesia antagonized (0.5 mg/kg Flumazenil, Siegfried Hameln, Hameln, Germany, and 2.5 mg/kg Atipamezole hydrochloride, Pfizer, Freiburg, Germany, i.p.) and the animals were transferred back into their cages. The animals were placed for 1.5 h in a neonatal incubator (IC8000, Draeger, Luebeck, Germany) with controlled air temperature (35°C) and ambient humidity (35%) to maintain constant body temperature and avoid hypothermia. The experimenter performing the CCI surgery was blinded to the treatment. A separate experimenter, also blinded to treatment, performed tissue preparations.

### Drug Preparation

WY-14643 (Tocris Bioscience, Bristol, UK) was dissolved in DMSO, warmed in 37°C water-bath and diluted in 0.9% NaCl solution to 10% DMSO end-concentration. GW6471 (Tocris Bioscience, Bristol, UK) was also dissolved in 10% DMSO with 0.9% NaCl solution. As vehicle control 10% DMSO in 0.9% NaCl solution was administered i.p.. WY-14643 or vehicle was administered 30 min before CCI. In experiments with both substances GW6471 or vehicle was administered 60 min before CCI and WY-14643 or vehicle 30 min before CCI. The experimentator was blinded to treatment.

### Euthanasia and Tissue Preparation

The animals were reanaesthetized as described and killed by cervical dislocation. For the quantification of brain water content and further investigations the cerebellum was separated and the hemispheres were cut along the interhemispheric plane slightly modified as described before (Timaru-Kast et al., 2012). Both hemispheres were separated again in the middle of the contusion area and weighed to assess their wet weight. One half of each hemisphere was dried in a vacuum-centrifuge (Univapo 100 H, UniEquip, Planegg, Germany) for 48 h at 39°C to determine the dry weight. On the basis of the gravimetrical differences, brain water content was obtained by the following calculation: Hemispheric water content (%): (WW - DW)/WW × 100, where WW is the wet weight (g) and DW is the dry weight (g) of the brain hemispheres (Thal et al., 2009). The other half of the hemispheres were frozen in liquid nitrogen and stored at -80°C for tissue analysis.

### Statistical Analyses

Statistical tests were performed using SigmaPlot 12.5 including SigmaStat tools applying One-Way ANOVA multiple comparison versus control with Holm–Sidak method. Data sets showing either no normal distribution or equality of variances were analyzed using Kruskal–Wallis One-Way ANOVA Rank Test followed by an all pairwise multiple comparison procedure with Dunn’s Method. Results are given as mean ± standard deviation (SD), or if indicated, as standard error of mean (SEM). The level of statistical significance was set at *p* < 0.05, indicated with asterisk (^∗^).

## Results

### WY-14643 Inhibits Barrier Breakdown during OGD

**FIGURE 1:**
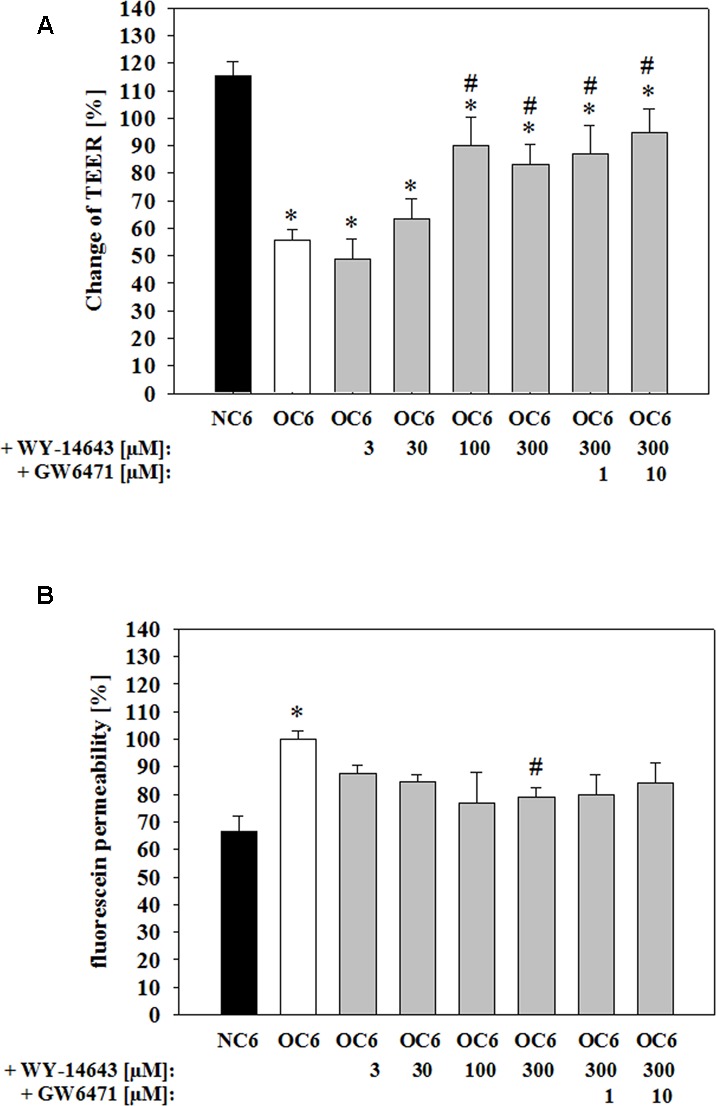
**Attenuation of barrier breakdown of cerebEND/C6 co-culture on Transwell inserts after 4 h oxygen/glucose deprivation (OGD) treatment by PPARα agonist WY-14463 and PPARα antagonist GW6471 – influence on transendothelial electrical resistance (TEER) (A)** or fluorescein permeability **(B)**. Statistical significance was labeled with ^∗^ vs. NC6 and ^#^ vs. OC6 (*p* < 0.05). Data are presented as means ± SD (*n* = 6–36).

At the beginning of the experiments TEER values were about 27.6 ± 2.1 Ω × cm^2^ (raw data differences of ∼80 Ωs between empty inserts and inserts with cells). After 4 h of OGD treatment TEER was decreased to 56 ± 4% (mean ± SEM, *n* = 36, *p* < 0.05), which was abolished by addition of PPARα agonist WY-14643 to 63 ± 7% (30 μM, *n* = 6, n.s.), 90 ± 10% (100 μM, *n* = 6, *p* < 0.05), and 83 ± 7% (300 μM, *n* = 15, *p* < 0.05) in a concentration dependent manner (**Figure 1A**). In concordance to this, permeability of paracellular marker fluorescein was increased by OGD treatment from 66.6 ± 5.8% (=2.03 ± 0.17 × 10^-5^ cm/s) to 100.0 ± 3.2% (mean ± SEM, *n* = 27, *p* < 0.05). Application of WY-14643 during OGD reduced fluorescein permeability to 84.6 ± 2.6% (30 μM, *n* = 6, n.s.), 76.7 ± 11.5% (100 μM, *n* = 6, n.s.), and 78.9 ± 3.6% (300 μM, *n* = 15, *p* < 0.05) (**Figure 1B**). Notably, addition of PPARα antagonist GW6471 did not reverse the BBB stabilizing effects of WY-14643. Therefore, it was verified whether WY-14643 activated PPARα in our model. Addition of 300 μM WY-14643 induced translocation of PPARα to the nucleus (Supplementary Figure S1). Moreover, mRNA expression of PPARα was decreased by OC6 and was further reduced by 300 μM WY-14643. The effect of WY-14643 on PPARα expression was blocked by 10 μM GW6471, whereas mRNA of PPARγ was upregulated by OC6 and not affected by addition of WY-14643 (Supplementary Table S3). These data indicated that WY-14643 induced PPARα activation in our model. In summary, WY-14643 reduced OGD-induced barrier damage significantly, but probably not by mechanisms dependent on the transcriptive activity of PPARα.

### Effects of WY-14643 on Tight Junction Proteins after OGD

Barrier functionality is linked to the expression and localization of tight junction proteins (Witt et al., 2003; Jiao et al., 2011). In order to investigate effects of WY-14643 on tight junction proteins, mRNA levels were measured by qPCR, and protein expression and distribution was determined by western blotting. As recently shown, 4 h of OC6-treatment decreased mRNA expression of claudin-5, ZO-1, and occludin significantly (Neuhaus et al., 2014), but addition of 300 μM WY-14643 did not change mRNA expression of any of the investigated tight junction proteins (claudin-3, claudin-5, claudin-12, occludin, and ZO-1) after 4 h of OC6-treatment (data not shown).

**FIGURE 2:**
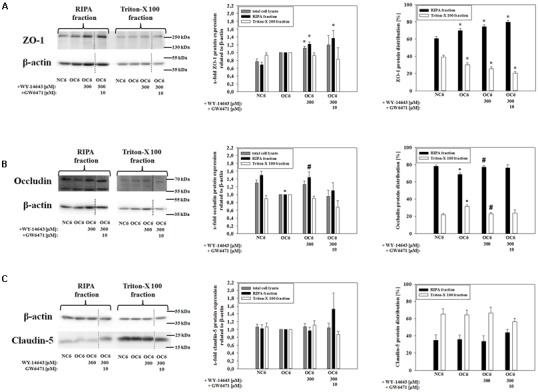
**Influence of WY-14643 and PPARα antagonist GW6471 on effects of OC6 treatment on the protein expression of tight junction proteins ZO-1, occludin and claudin-5 of cerebEND cells.** OC6 treatment of cerebENDs accords to 4 h OGD treatment with medium supernatants derived from 4 h OGD-treated C6 cells, whereas NC6 treatment means cerebEND cells incubated for 4 h under normoxic conditions with medium supernatants of 4 h normoxic treated C6 cells. Results for ZO-1 **(A)**, occludin **(B)** and claudin-5 **(C)** were displayed. Densitometric analyzed protein expression of total cell lysates were compared to RIPA and triton-X 100 fractions. Moreover, protein distributions [%] between RIPA and triton-X 100 fractions were presented. Values were related to β-actin and according representative western blot images are depicted. Dotted lines indicated cuts of images of the same blot due to presentation reasons of selected bands. Statistical significance was labeled with ^∗^ vs. NC6 and ^#^ vs. OC6 (*p* < 0.05). Data are presented as means ± SEM (*n* = 3–8).

Western blotting analysis revealed a slight, but not significant upregulation of ZO-1 protein by OC6-treatment in comparison to the normoxic control (0.77 vs. 1.00-fold, **Figure 2A**). Addition of 300 μM WY-14643 during OC6 significantly increased total ZO-1 protein to 1.12 ± 0.05-fold, and the addition of GW6471 did not reverse this effect. In order to test for a change in cellular localization after the treatments, cells were lysed first in triton-X 100 and residual cell components were dissolved in RIPA buffer. The fraction soluble in triton-X 100 was enriched with plasma membrane proteins, whereas the fraction insoluble in triton-X 100, so-called RIPA fraction, contained mainly cytoskeleton/cytosolic proteins allowing to investigate the movement of proteins between these two fractions. For example, a significant enrichment of plasma membrane marker Na^+^/K^+^-ATPase was shown in the triton-X 100 fraction, whereas cytoskeleton protein β-actin was mainly found in the RIPA-fraction (see Supplementary Figure S2).

In case of ZO-1, the distribution between the triton-X 100 fraction and the RIPA-fraction was changed. OC6, OC6+WY-14643, and OC6+WY14-643+GW6471 stepwise reduced the amount of ZO-1 in the triton-X 100 fraction. Together with the expression data, it was shown that the amount of ZO-1 in the RIPA-fraction was significantly increased by WY-14643 during OC6 by 22% to 1.22 ± 0.06-fold, whereas the triton-X fraction remained unaffected. Addition of 10 μM GW6471 to WY-14643 during OC6 treatment did not change the effects of WY-14643 again. In case of occludin (65 kDa), its expression decreased in the RIPA-fraction from 1.49+0.11-fold (NC6) to 1.00-fold (OC6) due to the OC6-treatment. This change in expression was again prevented by addition of 300 μM WY-14643 (1.44 ± 0.14-fold). Interestingly, the effect of WY-14643 on occludin expression was reversed by PPARα antagonist GW6471 to 1.10 ± 0.20 indicating a PPARα dependency. Moreover, OC6 treatment resulted in a movement of occludin into the triton-X 100 fraction from 21.8 ± 1.5% (NC6) to 31.5 ± 1.9% (OC6), which was blocked by WY-14643 (22.8 ± 1.5%), but not reversed by GW6471 (23.8 ± 3.6%) (mean ± SEM, *n* = 3–6, **Figure 2B**). Based on these results, it could be assumed that occludin expression was PPARα dependent, but the protein distribution was PPARα independent. Protein expression of claudin-5 was not regulated either by OC6 nor by the addition of WY-14643 during OC6 treatment in comparison to the NC6 control (**Figure 2C**). In summary, changes of tight junction proteins by WY-14643 were only found on the protein level. The redistribution of tight junction proteins might play an important role for the observed barrier breakdown.

### Effects of WY-14643 on the Phosphorylation of MAP Kinases after OGD

**FIGURE 3:**
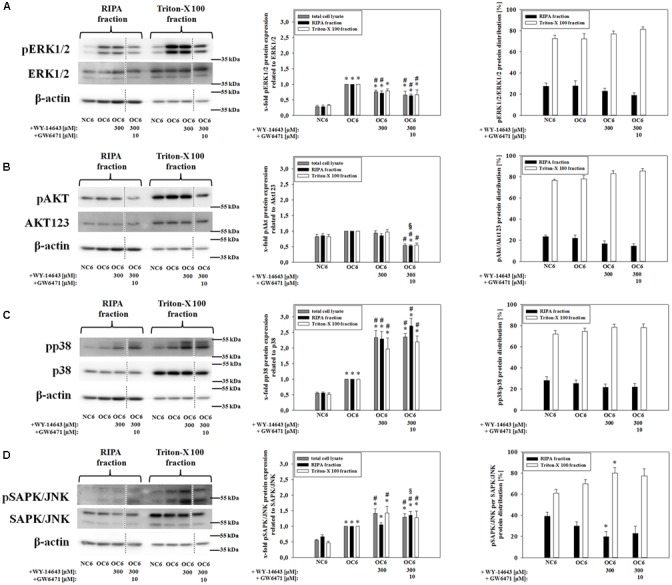
**Influence of WY-14643 and PPARα antagonist GW6471 on effects of OC6 treatment on phosphorylation ratios of MAP kinases ERK1/2, Akt, p38, and SAPK/JNK of cerebEND cells.** OC6 treatment of cerebENDs accords to 4 h OGD treatment with medium supernatants derived from 4 h OGD-treated C6 cells, whereas NC6 treatment means cerebEND cells incubated for 4 h under normoxic conditions with medium supernatants of 4 h normoxic treated C6 cells. Results for ERK1/2 **(A)**, Akt **(B)**, p38 **(C)**, and SAPK/JNK **(D)** were displayed. Densitometric analyzed protein expression of total cell lysates were compared to RIPA and triton-X 100 fractions. Moreover, protein distributions [%] between cytoskeleton (RIPA) and membrane enriched fractions (triton-X 100) were presented. Values were related to β-actin and according representative western blot images are depicted. Dotted lines indicated cuts of images of the same blot due to presentation reasons of selected bands. Statistical significance was labeled with ^∗^ vs. NC6, ^#^ vs. OC6 and ^§^ vs. OC6+WY (*p* < 0.05). Data are presented as means ± SEM (*n* = 3–6).

Tight junction protein localization is dependent on the level of intracellular calcium and is associated with their phosphorylation status (Fischer et al., 2005; Minshall and Malik, 2006; Srivastava et al., 2013). These parameters are linked to the activation of kinases which could be deduced from the protein expression ratio of phosphorylated kinase per total kinase. Therefore, effects of 300 μM WY-14643 on the phosphorylation ratio of MAP kinases – known for their role during cerebral ischemia (Ferrer et al., 2003; Yuen et al., 2014) – during OGD were investigated. The amount of phosphorylation of all four investigated kinases ERK1/2, Akt, p38, and SAPK/JNK in cerebEND cells were upregulated by 4 h of OC6 treatment (ERK1/2: from 0.28 to 1.00-fold, Akt: from 0.82 to 1.00-fold, p38: from 0.55 to 1.00-fold, and SAPK/JNK: from 0.55 to 1.00-fold), which was only for Akt not statistically significant (**Figure 3**). Addition of WY-14643 during OC6 treatment significantly reduced phosphorylation of ERK1/2 from 1.00 to 0.75-fold, but increased phosphorylation of p38 and SAPK/JNK to 2.33- and 1.42-fold, respectively. Addition of 10 μM GW6471 did not reverse the effects of WY-14643 on ERK1/2 or p38, it increased the phosphorylation of SAPK/JNK only in the RIPA-fraction and reduced the pAkt/Akt ratio for all fractions to 0.54-, 0.52-, and 0.55-fold. Interestingly, administration of 300 μM WY-14643 led to an increased amount of pSAPK/JNK only in the Triton-X 100 (membrane) fraction. In summary, addition of PPARα agonist WY-14643 modulated the phosphorylation grade of ERK1/2, p38 and SAPK/JNK that was only reversed in the RIPA-fraction of SAPK/JNK by PPARα antagonist GW6471.

### WY-14643 Is able to Inhibit Activity of Proteasomal Enzymes

**FIGURE 4:**
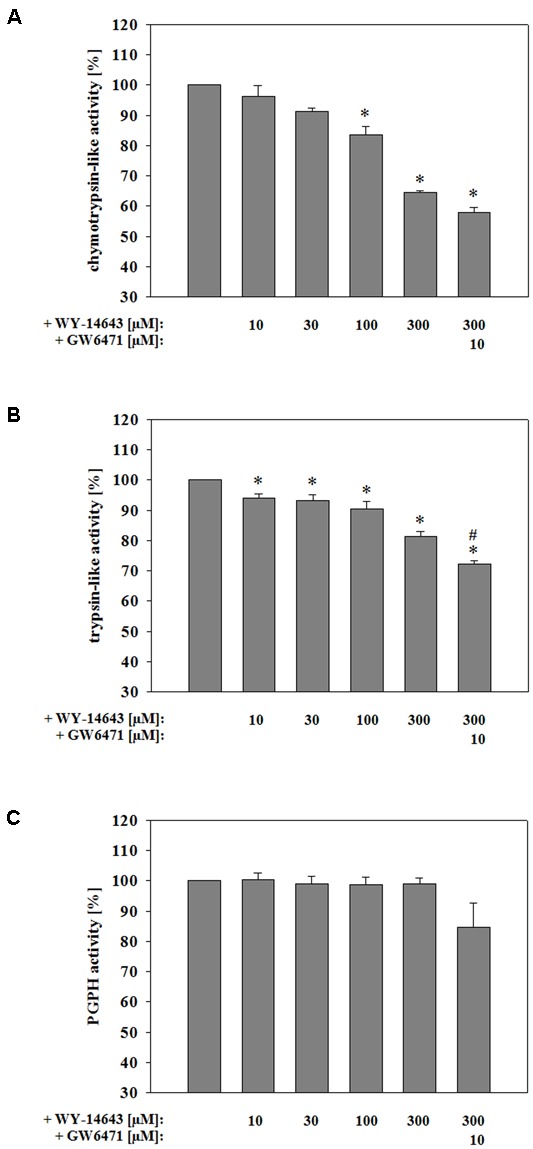
**Influence of WY-14643 on proteasomal chymotrypsin-like (A)**, trypsin-like **(B)**, and peptidylglutamyl-peptide hydrolyzing activity (PGPH) **(C)** activity after isolation of the enzymes from cerebEND cells. Statistical significance was labeled with ^∗^ vs. NC6 and ^#^ vs. OC6 (*p* < 0.05). Data are presented as means ± SEM (*n* = 3–4).

Recent work showed that blockade of the proteasome could contribute to the stabilization of the BBB during TBI as well as stroke (Kleinschnitz et al., 2011; Thal et al., 2013). Therefore, effects of WY-14643 on proteasomal activity was investigated. Interestingly, no significant changes were found in proteasomal activity when 300 μM WY-14643 were added during 4 h of OC6 treatment followed by enzyme isolation and activity measurement. Thus, in order to test whether WY-14643 inhibition of proteasomal enzymes could be reversible and was abolished by the enzyme isolation procedure, WY-14643 was added after isolation of proteasomal enzymes of cerebEND cells. This procedure led to a significant blockade of chymotrypsin-like (300 μM WY-14643: 64.6 ± 0.59%) and trypsin-like activity (300 μM WY-14643: 81.2 ± 1.8 %) by WY-14643 in a concentration dependent manner (**Figure 4**), whereas PGPH activity was not altered. Addition of 10 μM of PPARα antagonist GW6471 did not reverse effects by WY-14643, it even further blocked the activity of chymotrypsin-like (300 μM WY-14643+10 μM GW6471: 57.9 ± 1.7%) and trypsin-like (300 μM WY-14643+10 μM GW6471: 72.3 ± 1.1%) proteasomal activity significantly. In summary, WY-14643 could inhibit proteasomal activity that was not reversed by PPARα antagonist GW6471.

### WY-14643 Reduces Activity of Tissue-Plasminogen Activator (t-PA) after OGD

**FIGURE 5:**
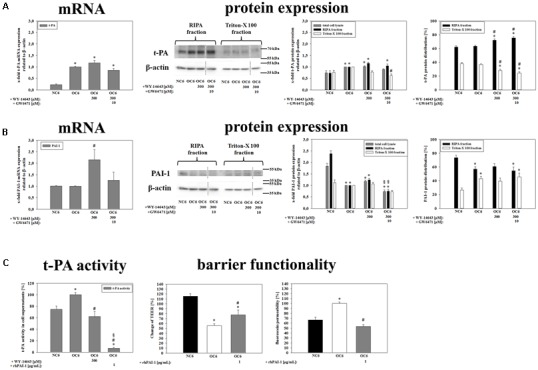
**Influence of WY-14643 on t-PA and PAI-1 expression and activity in the blood-brain barrier (BBB) *in vitro* ischemia model.** OC6 treatment of cerebENDs accords to 4 h OGD treatment with medium supernatants derived from 4 h OGD-treated C6 cells, whereas NC6 treatment means cerebEND cells incubated for 4 h under normoxic conditions with medium supernatants of 4 h normoxic treated C6 cells. Results on mRNA as well as protein expression of t-PA **(A)** and PAI-1 **(B)** of cerebEND cells after OC6 treatment were displayed. Effects of WY-14643 on t-PA activity and of rhPAI-1 on barrier functionality were shown in **(C)**. Densitometric analyzed protein expression of total cell lysates were compared to RIPA and triton-X 100 fractions. Protein distributions [%] between cytoskeleton (RIPA) and membrane enriched fractions (triton-X 100) were presented. Values were related to β-actin and according representative western blot images are depicted. Dotted lines indicated cuts of images of the same blot due to presentation reasons of selected bands. Statistical significance was labeled with ^∗^ vs. NC6, ^#^ vs. OC6 and ^§^ vs. OC6+WY (*p* < 0.05). Data are presented as means ± SEM (mRNA: *n* = 4–8, protein expression: *n* = 3–6, t-PA activity: *n* = 8–10, and barrier functionality: *n* = 12–36).

It is rather well established that MMPs and t-PA contribute to BBB breakdown during cerebral ischemia (Pfefferkorn and Rosenberg, 2003; Adibhatla and Hatcher, 2008). Therefore, firstly effects of WY-14643 on mRNA expression of MMP2, MMP3, and MMP9, and of their endogenous inhibitors TIMP1 and TIMP3 were investigated. Three hundred micrometer WY-14643 decreased expression of MMP3 to 0.75-fold and TIMP1 to 0.68-fold after 4 h OC6 treatment. Addition of 10 μM GW6471 did not reverse effects of WY-14643, it further significantly decreased TIMP1 expression to 0.48-fold and increased TIMP3 expression from 1.11-fold (300 μM WY-14643 during OC6) to 1.37-fold. In concordance with the concomitant decrease of MMP3 and TIMP1 WY-14643 did not reduce total MMP-activity after 4 h of OC6 treatment. This indicated a minor role of MMP-activity for the beneficial effects of WY-14643 to prevent BBB breakdown *in vitro* (Supplementary Table S3). Then, expression of t-PA and its endogenous inhibitor PAI-1 was analyzed. mRNA expression of t-PA was significantly upregulated from 0.22 ± 0.04 (NC6) to 1.00-fold by OC6 treatment, however, WY-14643 addition showed no effect on t-PA mRNA expression (**Figure 5**). Western blotting confirmed mRNA expression results. OC6 treatment significantly upregulated protein expression of t-PA on the protein level in cerebENDs from 0.74 ± 0.09 to 1.00-fold, whereas addition of WY-14643 had no effects on total protein expression. Interestingly, application of 300 μM WY-14643 resulted in a significant redistribution of t-PA toward the RIPA (cytosol/cytoskeleton) fraction which was still consistent after addition of GW6471. In case of PAI-1, 4 h of OC6 treatment did not regulate mRNA expression, but application of 300 μM WY-14643 significantly upregulated PAI-1 from 1.00-fold (OC6) to 2.15 ± 0.44-fold. This upregulation was abolished by addition of 10 μM GW6471 indicating a PPARα dependent effect. Western blotting confirmed a slight PAI-1 upregulation by WY-14643 from 1.00 to 1.17 ± 0.06-fold (n.s.), which was significantly reduced by GW6471 to 0.74 ± 0.07-fold. Interestingly, OC6 treatment revealed a substance independent redistribution of PAI-1 toward the triton-X 100 (membrane) fraction. Moreover, OC6 led to a significant protein reduction of PAI-1 in cerebEND cells from 1.84 ± 0.12-fold (NC6) to 1.00-fold (OC6) indicating a significant release into the growth medium. Therefore, t-PA activity of cell culture medium supernatants was measured after the treatments. According to expression results of t-PA and PAI-1, OC6 treatment increased t-PA activity in cell supernatants from 74.7 ± 5.9% (NC6) to 100 ± 3.9% (OC6) which was reduced by WY-14643 to 62.1 ± 8.7%. Data suggested a major role of upregulated PAI-1 for WY-14643 mediated barrier stabilization. Addition of 1 μg/mL rhPAI-1 decreased t-PA activity in supernatants to 6.9 ± 1.6%. Moreover, 1 μg/mL rhPAI-1 blocked barrier breakdown significantly. In detail, decrease of TEER by OC6 treatment was attenuated from 55.8 ± 3.6% to 77.7 ± 9.9%, and fluorescein permeability was reduced from 100.0 ± 8.8% (OC6) to 53.3 ± 3.8% by addition of 1 μg/mL rhPAI-1 in comparison to 68.8 ± 5.7% of the normoxia control NC6. In summary, data suggested that upregulation of PAI-1 and subsequent deceased t-PA activity could be one major mechanism of the blockade of BBB breakdown by PPARα agonist WY-14643.

### WY-14643 Reduces Brain Edema Formation after Traumatic Brain Injury

**FIGURE 6:**
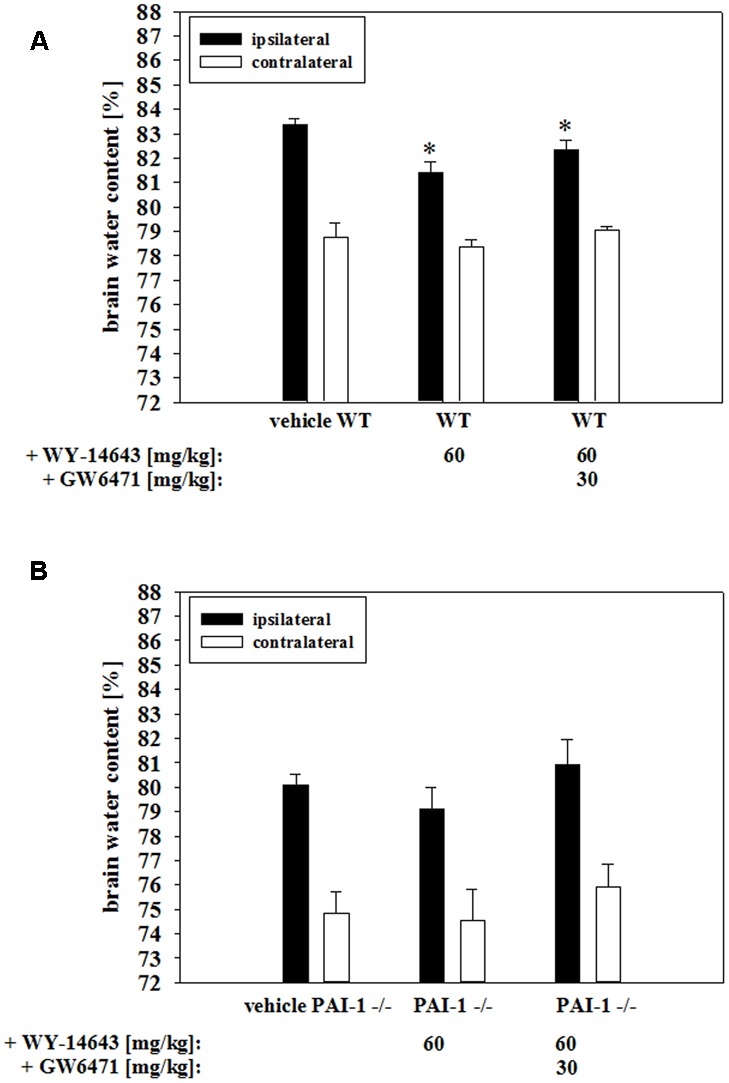
**Influence of WY-14643 on brain water content [%] 24 h after controlled cortical impact in a mouse traumatic brain injury model.** 60 mg/kg body weight of WY-14643 reduced the increase of brain water content in the ipsilateral brain hemisphere of wild type C57Bl/6 mice significantly. Addition of PPARα antagonist GW6471 did not reverse the beneficial effects of WY-14643 **(A)**. Administration of 60 mg/kg body weight WY-14643 did not decrease brain water content of PAI-1 knockout mice (PAI-1^-/-^) after TBI. **(B)**. Statistical significance was labeled with ^∗^ (*p* < 0.05). Data are presented as means ± SEM (wild type: *n* = 6–12; PAI-1^-/-^: *n* = 6–7).

In order to test the *in vivo* relevance of the found BBB stabilizing properties of WY-14643 during OGD, effects of addition of WY-14643 were investigated in a controlled cortical impact mouse model of TBI. This model comprises a significant cerebral ischemic component. Brain edema formation was measured as a major indicator of TBI after 24 h of the impact. The injury significantly increased brain water content in the vehicle control group about 4.61 ± 0.54% (mean ± SEM, *n* = 12, from 78.74 ± 0.62% in the contralateral hemisphere to 83.35 ± 0.26% in the ipsilateral hemisphere, **Figure 6A**). Administration of 60 mg/kg body weight of WY-14643 reduced the increased water amount significantly to 3.06 ± 0.25% (contralateral: 78.34 ± 0.34%, ipsilateral: 81.40 ± 0.45%, mean ± SEM, *n* = 12). Similar to BBB functionality *in vitro* data, combined treatment of 60 mg/kg body weight WY-14643 with 30 mg/kg body weight GW6471 did not reverse sole effects of WY-14643 in a significant manner. In this group, difference of water content between ipsilateral (82.36 ± 0.37%, mean ± SEM, *n* = 6) and contralateral hemisphere (79.04 ± 0.16%, mean ± SEM, *n* = 6) after TBI was 3.32 ± 0.36%. In order to verify the *in vivo* role of upregulated PAI-1 by WY-14643 found in the BBB *in vitro* model, effects of WY-14643 on edema formation in the same mouse TBI model using PAI-1 knockout (PAI-1^-/-^) mice were investigated. Addition of 60 mg/kg body weight WY-14643 decreased the increase of brain water content from 5.27 ± 0.59% (mean ± SEM, *n* = 7, ipsilateral: 80.08 ± 0.44%, contralateral: 74.81 ± 0.93%) to 4.52 ± 0.95% (mean ± SEM, *n* = 6, ipsilateral: 79.08 ± 0.92%, contralateral: 74.56 ± 1.26%, **Figure 6B**) in a non-significant manner and addition of GW6471 showed also no effect. In summary, these data revealed that WY-14643 was able to decrease edema formation in a TBI model which was not only PPARα dependent. Moreover, animal experiments with PAI-1^-/-^ mice supported the hypothesis about participation of PAI-1 in WY-14643 mediated effects. These *in vivo* data were in concordance with *in vitro* BBB results suggesting contribution of PPARα independent mechanisms as well as PPARα dependent PAI-1 regulation to multifaceted effects of WY-14643 during cerebral ischemia.

## Discussion

In seventy percent of brain trauma cases the neurovascular unit is compromised and the extent of neurovascular permeability correlates with that of cortical dysfunction (Tomkins et al., 2011). Moreover, the disruption of the neurovascular unit is linked to several long-term consequences of TBI such as Alzheimer’s disease and post-traumatic epilepsy (Shlosberg et al., 2010; Sashindranath et al., 2012). Cerebral ischemia or hypoxia during TBI induces leakage of the BBB which subsequently contributes to the development of cerebral edema. The formation of cerebral edema is one of the main factors for the high mortality and morbidity after TBI. Therefore, the preservation of the functionality of the neurovascular unit after TBI seems to be a promising strategy to significantly improve the clinical outcome.

In this context, PPARs have been considered as new pharmacological targets in cerebral ischemic insults (Bordet et al., 2006). Treatments with either fenofibrate, WY-14643 or glitazones reduced adverse outcomes (Escher and Wahli, 2000; Gautier et al., 2015). For example, administration of fenofibrate after TBI led to improved neurological scores, reduced cerebral edema, lesion volume and ICAM-1 expression in a rat model. In the same animal model fenofibrate acted also in an antiinflammatory and antioxidative manner (Besson et al., 2005; Chen et al., 2007). Similar effects have been found in stroke models. PPARα agonists enhanced vascular function and were neuroprotective by modulating inflammatory, immune and oxidative pathways. They decreased cerebral infarct volume, microglial activation, neutrophil infiltration and improved synaptogenesis, neurogenesis, neurorepair, motor function (short-term effect), cognitive post-stroke consequences and angiogenesis. Antioxidative and antiinflammatory properties of PPARα agonists were shown by increased superoxid-dismutase and decreased NFkb and AP-1 activity. Effects of PPARα agonists on the brain endothelium confirmed that the BBB significantly contributes to the disease progression. For example, PPARα agonists improved acetylcholine induced vascular relaxation by nitric oxide and enhanced cerebral blood flow in the ischemic penumbra, but also decreased superoxide generation and inhibited the upregulation of adhesion molecules ICAM-1 and VCAM-1 (Staels et al., 1998; Delerive et al., 1999; Escher and Wahli, 2000; Von Knethen and Brüne, 2001; Cunard et al., 2002a,b; Deplanque et al., 2003; Bordet et al., 2006; Ouk et al., 2009, 2014; Guo et al., 2010). Moreover, experiments with Apo-E deficient mice proved that fenofibrate could also act in a lipid/glucose metabolism independent manner, since the infarct size was still reduced by the PPARα agonist after these mice developed hyperglycemia (Deplanque et al., 2003). In addition, PPARα agonists induced protective effects against ischemia/reperfusion damage in peripheral organs including heart, kidney, and intestine (Sivarajah et al., 2002; Wayman et al., 2002; Yue et al., 2003; Cuzzocrea et al., 2004).

However, although previous studies showed that the brain endothelium and its permeability was affected by PPARα treatments during cerebral ischemic insults, detailed information about underlying mechanisms are still missing. Mysiorek et al. (2009) reported that fenofibrate was able to block BBB breakdown in an *in vitro* OGD model in a PPARα dependent manner, because brain endothelial cells isolated from PPARα knockout mice were not susceptible for that treatment. They further showed that fenofibrate acted directly on brain endothelial cells, but they have not found any PPARα target to explain the observed effects (Mysiorek et al., 2009). Therefore, the aim of the present study was to find ischemia relevant mechanisms modulated by PPARα agonists that could cause the protection of the BBB. For our studies, it was decided to use PPARα agonist WY-14643. In a previous study WY-14643 decreased the infarct volume in a MCAO model of permanent focal cerebral ischemia which was not found in PPARα knockout mice (Inoue et al., 2003). Moreover, WY-14643 reduced tissue damage after ischemia/reperfusion in liver, heart, and gut, but also increased angiogenesis in zebrafish (Wayman et al., 2002; Cuzzocrea et al., 2004; Okaya and Lentsch, 2004; Bulhak et al., 2009; Nemčeková et al., 2013; Rizvi et al., 2013). In the present study the comprehensively validated BBB *in vitro* OGD model based on a co-culture of mouse brain endothelial cerebENDs cells and rat glioma C6 cells was used to investigate the effects of WY-14643 (Neuhaus et al., 2014).

Similarly to the results of Mysiorek et al. (2009) the addition of a PPARα agonist – in our case WY-14643 – inhibited BBB barrier breakdown *in vitro* by OGD. This was shown by TEER and fluorescein permeability data. Surprisingly, PPARα antagonist GW6471 was not able to reverse WY-14643 mediated protection of barrier tightness. Paracellular tightness is linked to the expression and localization of tight junction proteins (Witt et al., 2003; Jiao et al., 2011). Interestingly, WY-14643 did not prevent the decrease of mRNA levels of tight junction proteins indicating no direct transcriptional PPARα dependent effects. On the contrary, western blotting results showed different effects on expression and protein distribution of tight junction proteins. Especially, effects on occludin were very interesting. Regulations of the RIPA-fraction by WY-14643 and GW6471 suggested a PPARα dependency, whereas the effects on protein distribution were according to TEER data indicating a PPARα independent occludin distribution. Corresponding to our data, Canfield et al. (2016) recently found changes in TEER that was related to altered tight junction protein localization. At the same time they did not detect a significant regulation of tight junction protein expression using a human BBB model based on endothelial cells differentiated from human induced pluripotent stem cells.

The localization status of tight junction proteins is dependent on their phosphorylated sites. Therefore, we investigated as next step the influence of WY-14643 on MAP kinases. Previous studies showed that kinases such as ERK1/2, Akt, p38, and SAPK/JNK could be involved during BBB breakdown. According to literature, phosphorylation of all four analyzed kinases increased after OC6 treatment in our model (Kyriakis and Avruch, 2001; Irving and Bamford, 2002; Cowan and Storey, 2003; Ferrer et al., 2003; Lee and Lo, 2003; Zhu et al., 2003; Collino et al., 2006; Zhang et al., 2007; Wallace et al., 2012; Yuen et al., 2014). Interestingly, WY-14643 decreased the phosphorylation of ERK1/2, but increased phosphorylation of p38 and SAPK/JNK indicating a complex regulatory network which should be decoded in future studies. Recent work revealed that inhibition of proteasomal activity could contribute to decelerated degradation of proteins relevant for tight junction protein expression (Kleinschnitz et al., 2011; Thal et al., 2013). Therefore, proteasomal inhibitory properties of WY-14643 were tested and indeed it was found for the first time that WY-14643 was capable of blocking proteasomal activity. However, the addition of proteasome inhibitors itself were not able to prevent BBB breakdown or tissue damage. It was suggested that the less degraded glucocorticoid receptor had to be still activated in order to induce expression of occludin in the murine BBB under ischemic conditions (Kleinschnitz et al., 2011). If and how this pathway is modulated by WY-14643 and plays a role in our model could be elucidated in further studies. In general, the relevance of the modulation of MAP kinases and proteasomal enzymes by PPARα and PPARγ for BBB stabilization were confirmed in previous studies concerning HIV mediated BBB breakdown (Huang et al., 2009, 2011, 2014).

One major finding of this study was that we were able to show for the first time that WY-14643 increased PAI-1 expression of brain endothelial cells after OC6 treatment and that this was associated with a decrease of t-PA activity. On the one hand t-PA is used as a drug to lyse thrombi during stroke treatment, on the other hand it is known that t-PA contributes to BBB breakdown via MMP activation or cleavage of membrane receptors of the brain endothelium (Pfefferkorn and Rosenberg, 2003; Adibhatla and Hatcher, 2008). Recent publications underlined the possible importance of this finding. Fenofibrate therapy was successful in an acute phase stroke model with a thrombolysis-induced hemorrhage, where thrombolysis was induced by t-PA – treatment. Here, fenofibrate reduced the risk of hemorrhage after thrombolysis and decreased infarct volume, the stroke-induced vascular endothelium dysfunction, microglial activation and neutrophil infiltrations (Gautier et al., 2015). In this case, it could be speculated that fenofibrate increased PAI-1 expression leading to less t-PA mediated damage.

An interesting question is whether the amount of PAI-1 derived from brain endothelial cells would be enough to inhibit its breakdown during an ischemic insult. In this regard, it was published that WY-14643 upregulated PAI-1 in cells of other tissues such as cardiomyocyte-like cells or hepatocytes (Banfi et al., 2003; el Azzouzi et al., 2011). Moreover, it was reported that stroke-induced upregulation of acute phase proteins in the liver was inhibited by fenofibrate leading to decreased leukocytosis in the brain (Losey et al., 2015). Another study showed that fenofibrate treatment of salt-loaded spontaneously hypertensive stroke-prone rats reduced brain damage. In these animals an initially renal failure is linked to a cerebral ischemic insult. During the process they develop systemic inflammation followed by hypertension and proteinuria leading to end-organ injury (Gelosa et al., 2010). Altogether these data support the hypothesis that organ–organ interactions might play an important role for the beneficial effects of PPARα agonists to decrease adverse outcomes after cerebral ischemic insults. In this context, we also determined possible effects of WY-14643 on astrocyte mimicking glioma C6 cells being part of our co-culture model. Although it was published that fenofibrate acted mainly directly on brain endothelial cells to block BBB breakdown (Mysiorek et al., 2009), we found several targets such as t-PA, PAI-1, Angpt1, PDGFb, GDNF, ApoE, and TNFα in C6 cells which were regulated at the mRNA level by WY-14643 after OGD treatment (see Supplementary Table S4).

An important observation in our study was that several WY-14643 effects were not reversed by PPARα antagonist GW6471 indicating that for these effects no transcriptional activity of PPARα was necessary. Moreover, it could be speculated that WY-14643 at the effective concentrations (100–300 μM) activated also other targets than PPARα, since distinctly lower EC_50_ values (5.38 μM) were reported for WY-14643-mediated PPARα activation (Bernardes et al., 2013). To evaluate our *in vitro* data in an *in vivo* model, mice were subjected to CCI-TBI and treated with WY-14643 and GW6471 before. WY-14643 reduced edema formation significantly, which was also not inhibited by addition of PPARα antagonist GW6471. Western blotting of total brain samples revealed that similar targets (PAI-1, ZO-1, and ERK1/2) were affected by WY-14643 treatment *in vivo* as already found *in vitro* (data not shown). Moreover, experiments with PAI-1 knockout mice proved the relevance of PAI-1 for WY-14643 induced decrease of edema formation. It has to be mentioned that the CCI-TBI model also comprises tissue damage and inflammatory components which can influence edema formation, and that edema formation is only partly linked to BBB breakdown. However, we have chosen to analyze edema formation in order to evaluate whether the data obtained with a BBB *in vitro* OGD model could be predictive for a general parameter of CCI-TBI mediated damage.

In summary, we present several novel findings at the molecular level about how a PPARα agonist could prevent BBB damage in cerebral ischemic insults. Future studies might elucidate the underlying pathways to understand the processes in more detail and the data relevance for humans.

## Author Contributions

WN contributed to the study via its initial planning, experimental planning, collection of data, data analysis/interpretation and via the writing of the manuscript. TK contributed to the study via experimental planning, collection of data, data analysis/interpretation and via the writing of the manuscript. CG contributed to the study via experimental planning, collection of data and data analysis/interpretation. ST and CF contributed to the study via data analysis/interpretation and revision of the manuscript. AN contributed via the collection and analysis of data.

## Conflict of Interest Statement

The authors declare that the research was conducted in the absence of any commercial or financial relationships that could be construed as a potential conflict of interest.
