# Supplementary material for: Multifaceted Mechanisms of WY-14643 to Stabilize the Blood-Brain Barrier in a Model of Traumatic Brain Injury
Source: Front Mol Neurosci. 2017 May 26;10:149. doi: 10.3389/fnmol.2017.00149 (PMC5445138; doi:10.3389/fnmol.2017.00149)
Supplement: Supplementary file 1 [file Presentation_1.pdf]

Supplementary Material

Multifaceted mechanisms of WY-14643 to stabilize the blood-brain barrier during cerebral ischemia

Winfried Neuhaus*, Tobias Krämer, Anja Neuhoff, Christina Gölz, Serge C. Thal, Carola Y. Förster

*** Correspondence:** Winfried Neuhaus: winfried.neuhaus@ait.ac.at

Supplementary tables:

1ST: Taqman probes used for qPCR analysis

Target	Taqman® probe identification number
Abcb1a	Mm00440761_m1
Abcc4	Mm01226380_m1
Abcg2	Mm00496364_m1
Angiopoietin-2	Mm00545822_m1
β-actin	Mm01205647_g1
Caveolin-1	Mm00483057_m1
Claudin-1	Mm00516701_m1
Claudin-3	Mm00515499_s1
Claudin-4	Mm00515514_s1
Claudin-5	Mm00727012_s1
Claudin-12	Mm01316511_m1
Hif1a	Mm00468869_m1
ICAM-1	Mm00516023_m1
Kdr (VEGFR2)	Mm00440099_m1
Lrp1	Mm00464608_m1
MMP-2	Mm00439498_m1

MMP-3	Mm00440295_m1
MMP-9	Mm00442991_m1
Neuropilin-1	Mm00435379_m1
Occludin	Mm00500912_m1
Plat (t-PA)	Mm00476931_m1
Serpine1 (PAI-1)	Mm00435860_m1
Serpine F2	Mm00435868_m1
SOD	Mm01344233_g1
Tek (Tie-2)	Mm00443243_m1
Timp1	Mm00441818_m1
Timp3	Mm00441826_m1
Tjp1 (ZO-1)	Mm01320637_m1
Vegfa	Mm01281449_m1
Angpt1	Rn00585552_m1
ApoE	Rn04219668_g1
GDNF	Rn00569510_m1
PDGFb	Rn01502596_m1
Plat (t-PA)	Rn01482578_m1
Serpine1 (PAI-1)	Rn01481341_m1
TNFα	Rn01525859_g1

2ST: Antibodies used for western blotting and immunofluorescence microscopy

Target	Product number, Company	Species	Application dilution
Akt1/2/3	sc-8312, Santa Cruz	rabbit	1:200 for WB
pAkt	#4060, Cell Signaling TECHNOLOGY®	rabbit	1:100 for WB
Erk1/2	#9102, Cell Signaling TECHNOLOGY®	rabbit	1:200 for WB
phospho-Erk1/2	#9101, Cell Signaling TECHNOLOGY®	rabbit	1:200 for WB
p38	#9212, Cell Signaling TECHNOLOGY®	rabbit	1:1000 for WB
phospho-p38	#9211, Cell Signaling TECHNOLOGY®	rabbit	1:1000 for WB
SAPK/JNK	#9252, Cell Signaling TECHNOLOGY®	rabbit	1:200 for WB
phospho-SAPK/JNK	#9251, Cell Signaling TECHNOLOGY®	rabbit	1:50-1:100 for WB
Claudin-5	34-1600, Zymed®, Invitrogen	rabbit	1:200 for WB
Lamin-A	ab8980 [133A2], Abcam	mouse	1:100 for IF
Na^+/K^+-ATPase	sc-28800, Santa Cruz	rabbit	1:100 for WB
Occludin	71-1500, Zymed®, Invitrogen	rabbit	1:200 for WB
PAI-1	ab28207, Abcam	rabbit	1:100 for WB
PPARα	sc9000, Santa Cruz (H-98),	rabbit	1:100 for IF
t-PA	ab62763, Abcam	rabbit	1:150 for WB
ZO-1	40-2300, Zymed®, Invitrogen	rabbit	1:100 for WB
β-actin	A3854[$], SigmaAldrich	mouse	1:25,000 for WB
HRP-anti-mouse	LNA931V/AG, GE Healthcare UK ltd.		1:5000 for WB
HRP anti-rabbit	LNA934V/AG, GE Healthcare UK ltd		1:5000 for WB
anti-rabbit IgG Alexa Fluor 488	A21206, Invitrogen	donkey	1:200 for IF
anti-mouse IgG Alexa Fluor 594	A21203, Invitrogen	donkey	1:200 for IF

$: Peroxidase-linked antibody, usage of secondary antibodies was not applied

3ST: Influence of WY-14643 (300 µM) and combination with GW6471 (10 µM) on mRNA expression of PPARα, PPARγ, matrixmetalloproteinases MMP-2, -3 and -9, and TIMP-1 and TIMP-3, SOD1 and LRP1 of cerebEND cells; NC6 = cerebEND cells 4h normoxia with C6-medium, OC6 = cerebEND cells 4h OGD with C6-OGD medium. Data are presented as means ± SEM (n=4-8). Statistical significance was labelled with * versus NC6, #: significant versus OC6, §: significant to OC6+WY-14643 ($p<0.05$).

	**NC6**	**OC6**	**OC6 +WY-14643**	**OC6 + WY-14643 + GW6471**
**PPARα**	2.08 ± 0.38	1.00 ± 0.05*	0.76 ± 0.06*#	1.08 ± 0.10
**PPARγ**	0.75 ± 0.04	1.00 ± 0.06*	1.18 ± 0.02*#	1.05 ± 0.02*§
**MMP-2**	1.29 ± 0.08	1.00 ± 0.05*	0.93 ± 0.07*	0.92 ± 0.06*
**MMP-3**	1.24 ± 0.11	1.00 ± 0.09	0.75 ± 0.05*#	0.71 ± 0.05*#
**MMP-9**	0.83 ± 0.07	1.00 ± 0.12	0.92 ± 0.12	1.04 ± 0.13
**TIMP-1**	1.05 ± 0.05	1.00 ± 0.10	0.68 ± 0.03*#	0.48 ± 0.03*#§
**TIMP-3**	0.81 ± 0.01	1.00 ± 0.11	1.19 ± 0.01*	1.37 ± 0.02*#§
**SOD1**	1.23 ± 0.07	1.00 ± 0.03*	0.84 ± 0.05*#	0.86 ± 0.03*#
**LRP1**	1.66 ± 0.24	1.00 ± 0.02*	0.67 ± 0.02*#	0.45 ± 0.04*#§

4ST: Influence of WY-14643 (300 µM) on mRNA expression of t-PA, PAI-1, ANGPT1, PDGFb, GDNF, ApoE and TNFα of C6 cells; normoxia = C6 cells 4h normoxia, OGD = C6 cells 4h OGD. Data are presented as means ± SEM (n=6). Statistical significance was labelled with * versus normoxia, #: significant versus OGD ($p<0.05$).

	**normoxia**	**OGD**	**OGD +WY-14643**
**t-PA**	1.25 ± 0.04	1.00 ± 0.02*	1.24 ± 0.04#
**PAI-1**	0.84 ± 0.02	1.00 ± 0.02*	1.73 ± 0.11*#
**ANGPT1**	2.11 ± 0.16	1.00 ± 0.03*	1.89 ± 0.23#
**PDGFb**	1.16 ± 0.04	1.00 ± 0.01*	0.77 ± 0.02*#
**GDNF**	0.39 ± 0.01	1.00 ± 0.01*	0.82 ± 0.03*#
**ApoE**	0.84 ± 0.07	1.00 ± 0.03	0.71 ± 0.09#
**TNFα**	0.28 ± 0.03	1.00 ± 0.03*	1.84 ± 0.16*#

Supplementary figure SF1

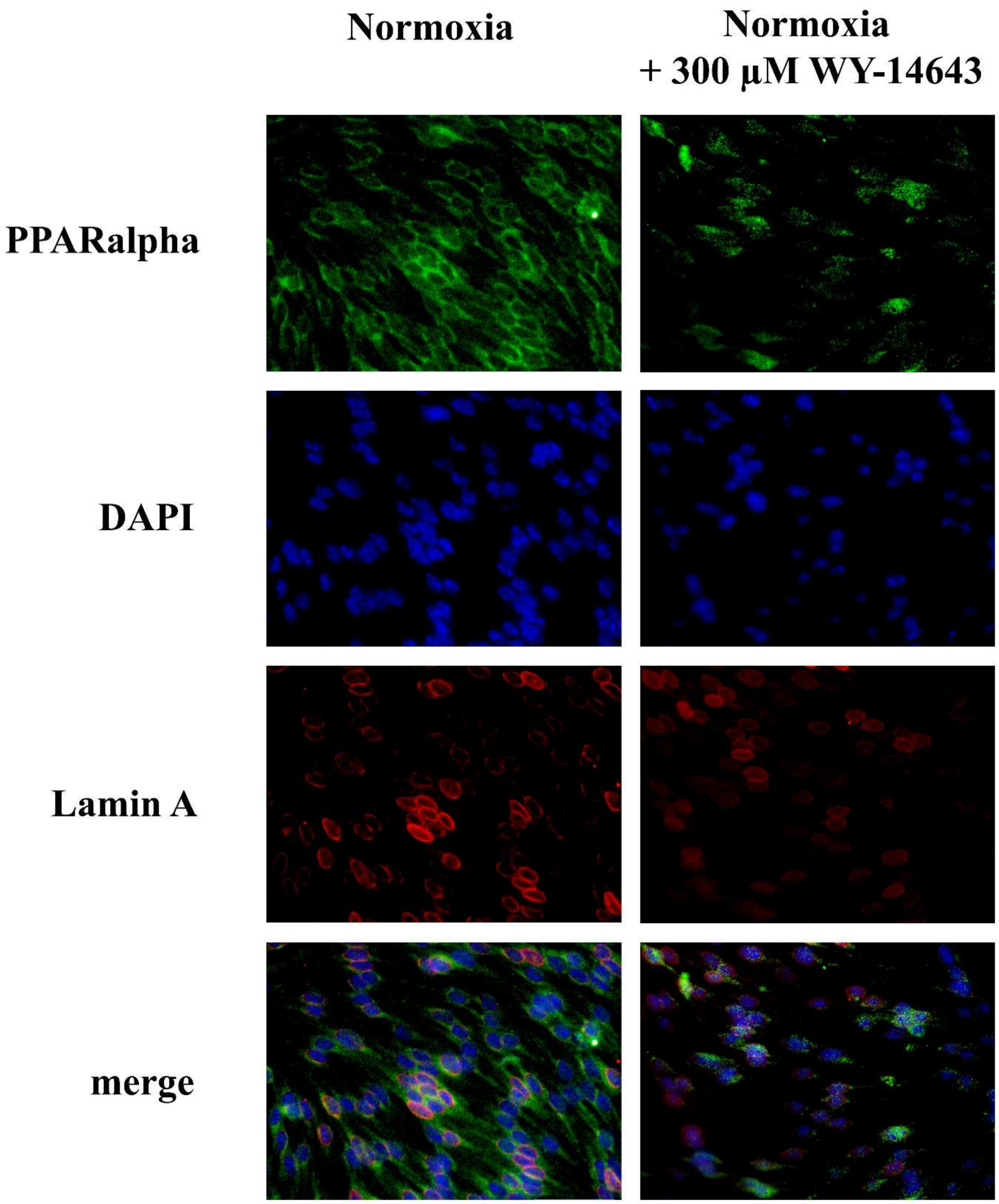

S1F: Immunofluorescence images of cerebEND cells treated with 300µM WY-14643 show that PPARalpha was translocated by WY-14643 into the nucleus area. PPARalpha was stained in green, DAPI (blue) to display DNA, nuclei pore protein lamin A was red.

Supplementary figure SF2

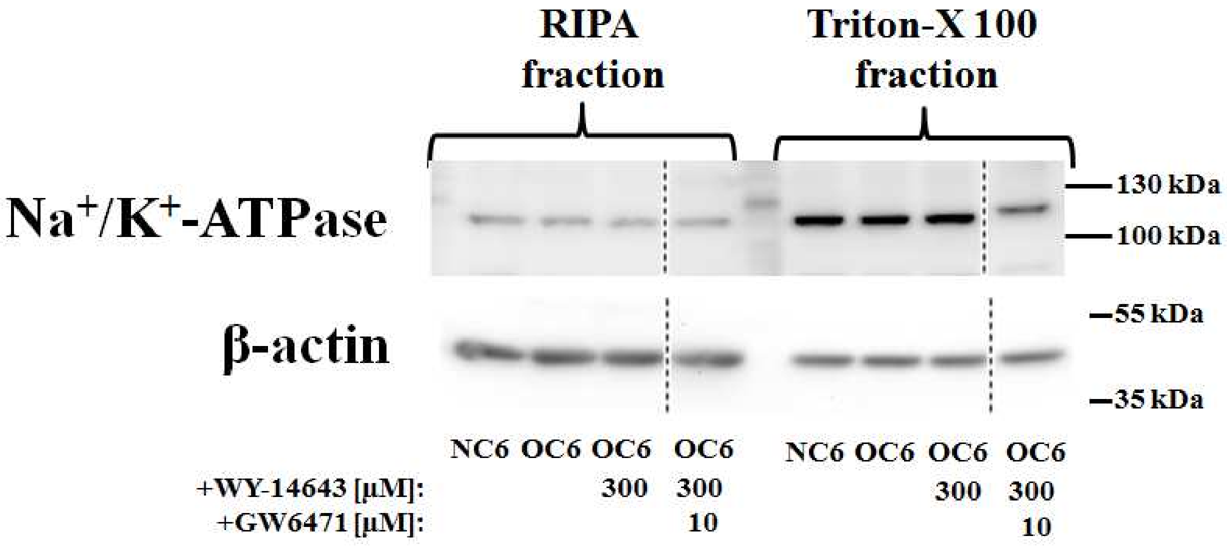

S2F: Influence of WY-14643 and PPARα antagonist GW6471 on effects of OC6 treatment on the protein expression of plasma membrane marker Na^+/K^+-ATPase of cerebEND cells. OC6 treatment of cerebENDs accords to four hours OGD treatment with medium supernatants derived from four hours OGD-treated C6 cells, whereas NC6 treatment means cerebEND cells incubated for four hours under normoxic conditions with medium supernatants of four hours normoxic treated C6 cells. Dotted lines indicated cuts of images of the same blot due to presentation reasons of selected bands.

Plasma membrane marker Na^+/K^+-ATPase was significantly stronger found in the triton-X 100 fraction indicating an enrichment of membrane proteins in the triton-X 100 fraction, whereas cytoskeleton protein β-actin was preferentially found in the RIPA-fraction.

Method description

Immunofluorescence micropscopy

Immunofluorescence microscopy was carried out as published recently (Neuhaus et al., 2014; Neuhaus et al., 2008; Neuhaus et al., 2012b). Applied primary and secondary antibodies could be found in supplementary table 2S. Images were generated by using a Biorevo Keyence BZ-9000 microscope and analyzed with the BZ-II Analyser software.
